# The Role of Adipokines between Genders in the Pathogenesis of Osteoarthritis

**DOI:** 10.3390/ijms251910865

**Published:** 2024-10-09

**Authors:** Alessio Economou, Ilenia Mallia, Antonella Fioravanti, Stefano Gentileschi, Francesca Nacci, Silvia Bellando Randone, Gemma Lepri, Serena Guiducci

**Affiliations:** 1 Rheumatology Unit, Department of Clinical and Experimental Medicine, University of Florence, 50134 Florence, Italy; francescanacci@icloud.com (F.N.); silvia.bellandorandone@unifi.it (S.B.R.); lepri.gemma@gmail.com (G.L.); serena.guiducci@unifi.it (S.G.); 2 Rheumatology Unit, Department of Medical Sciences, Surgery and Neurosciences, University of Siena, 53100 Siena, Italy; fioravanti7@virgilio.it (A.F.); stefano.gentileschi@unisi.it (S.G.)

**Keywords:** osteoarthritis, gender, sex, men, women, males, females, hormones, adipokines, synovial fluid

## Abstract

Osteoarthritis (OA) is a chronic, progressive, degenerative joint disease characterized by joint pain, stiffness, and limited movement. It presents significant intra- and inter-individual variability—in particular, between genders. Recent research has increasingly focused on the role of adipokines—especially leptin, adiponectin, and resistin—in the development of OA. Adipokines, peptide hormones primarily secreted by adipose tissue, are involved in crucial physiological processes related to metabolism and immunity. They can also impact bone and cartilage turnover by interacting with joint cells such as osteoblasts, osteoclasts, chondrocytes, and mesenchymal stem cells, thereby linking inflammation with bone cartilage homeostasis. This review aims to elucidate the structure and functions of various adipokines, their serum and synovial levels, and their association with clinical presentation and radiographic progression in OA patients, with a focus on differences between sexes. A narrative literature review was conducted using three databases specifically analyzing sex differences. OA patients generally show elevated serum and synovial levels of leptin, chemerin, and visfatin, as well as high plasma levels of resistin and visfatin. In contrast, synovial levels of adiponectin and omentin are reduced in OA patients compared to healthy individuals, with an inverse relationship to disease severity, suggesting a potential protective role. Resistin and leptin were positively correlated with pain severity and radiographic progression, while adiponectin’s role in OA remains controversial. Regarding sex differences, male OA patients exhibited higher serum levels of leptin, chemerin, and omentin compared to healthy controls, with a positive correlation to the BMI and estrogen levels, potentially explaining the sexual dimorphism observed in this condition. Studies on visfatin and lipocalin did not reveal significant differences in synovial or serum levels between the sexes. The role of resistin remains controversial. Adipokines influence the joint microenvironment and contribute to the progression of osteoarthritis (OA). However, the precise biological mechanisms are not yet fully understood due to the complex interactions between the metabolic, mechanical, and immune systems. Further research is needed to clarify their roles in OA and to identify targeted therapies for managing this degenerative disease.

## 1. Introduction

Osteoarthritis (OA) is a chronic, progressive, degenerative joint disease that can affect the articular cartilage, subchondral bone, ligaments, joint capsule, and synovium [1]. Any synovial joint (diarthrosis) can be involved, although the hips, knees, and small joints of the hands are the most commonly affected sites [2]. According to the 2021 Global Burden of Disease Study, the global prevalence of OA in 2020 was estimated at 7.6% (95% UI 6.8–8.4), affecting 595 million people (with a 95% uncertainty interval of 535–656 million). The total number of cases has risen since 1990, and the incidence rate is expected to increase by 2050, with projections indicating a 74.9% (59.4–89.9) rise in knee OA, 48.6% (35.9–67.1) in hand OA, 78.6% (57.7–105.3) in hip OA, and 95.1% (68.1–135.0) for other types [3]. OA prevalence increases with age, affecting the majority of individuals over 65 and leading to impaired mobility in the elderly [4].

Clinically, OA is characterized by joint pain, stiffness, and restricted movement, with occasional effusions and various degrees of local inflammation [5]. However, it is highly variable, ranging from an asymptomatic incidental finding to a significantly disabling disorder [6]. Several factors can influence disease progression, including person-related factors (such as age, gender, genetics, and body constitution) and joint-related factors (such as injuries, diseases, or abnormal joint loading), all interacting in complex ways [7].

Focusing on gender differences, studies have shown that the prevalence, incidence, and severity of OA differ between men and women. Men have a significantly lower risk of developing knee and hand OA, whereas women, particularly those over 55 years old, tend to experience more severe knee OA [8]. However, the prevalence of knee OA has also significantly increased among younger age groups (0–34 years, 35–39 years, and 40–44 years), reflecting an unexpected trend towards younger onset [9].

In clinical studies, women have reported higher levels of pain and severe functional limitations, even after surgical treatment. In contrast, men tend to achieve faster and better functional and physical recovery, regardless of the joint replaced. Caucasian women, in particular, experience poorer functional outcomes, especially in knee replacements [10].

Several factors contribute to making OA a distinct condition between the sexes. First, sex differences are evident in the microscopic anatomical features of the affected bones. Li et al. analyzed the femoral heads of 110 OA patients and found that remodeling processes increase with age in subchondral trabecular bone (STB) in men but not in women, while, in deeper trabecular bone (DTB), the opposite happens, with increased remodeling in women but not men. However, no correlation between bone microarchitecture and age was found in either sex [11].

The clinical involvement of sex hormones has also been clearly demonstrated: estrogens reduce pain in a dose-dependent manner, while testosterone decreases sensitivity to chronic pain [12]. Additionally, sex hormones and their receptors have protective effects against articular cartilage degradation, which may partially explain the greater progression of OA in women after menopause [13]. It is well established that women have thinner articular cartilage [14] and lose it at four times the annual rate of men in the proximal tibia and three times the rate in the patella [15].

### Obesity, Metabolic Syndrome and Adipokines in Osteoarthritis

Obesity is one of the most significant and modifiable risk factors for osteoarthritis (OA), characterized by the excessive accumulation of adipose tissue, particularly in weight-bearing joints such as the knees and hips [16]. It is well established that adipose tissue is not merely a passive storage depot but an active endocrine organ that secretes pro-inflammatory cytokines, hormones, and other signaling molecules, collectively known as adipokines (produced by adipose tissue) or adipocytokines (primarily, but not exclusively, released from adipocytes) [17]. Adipokines regulate various essential physiological processes, such as metabolism and immunity, and imbalances in their levels can contribute to the development and progression of several pathological conditions, including insulin resistance, dyslipidemia, hypertension, and coronary artery disease [18].

**Figure 1:**
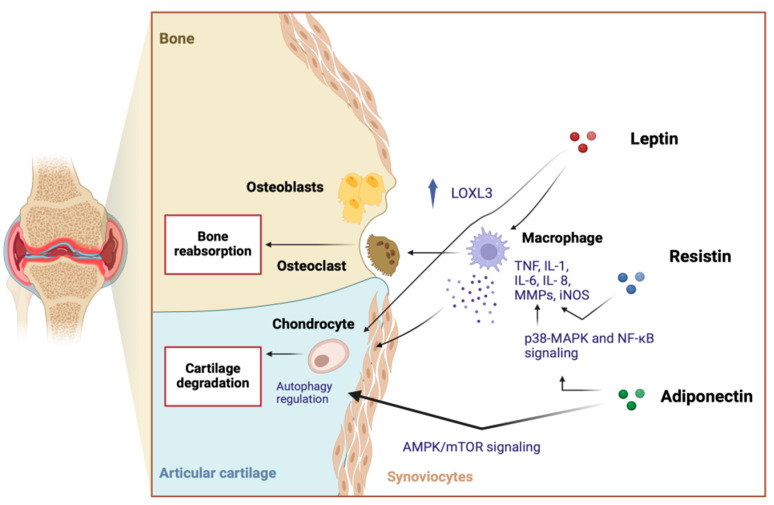
The role of the major adipokines in the pathogenesis of osteoarthritis, the main pathways influencing bone turnover and the joint microenvironment. Created with BioRender.com.

These proteins play a critical role in the onset and severity of OA, acting as mediators between adipose tissue and joint structures, influencing cartilage metabolism and bone structural changes through multiple mechanisms [19] Some adipokines, such as leptin, adiponectin, resistin, and visfatin, induce the production of inflammatory cytokines (e.g., IL-6 and TNF-α) within the joint, contributing to determine an inflammatory microenvironment [20]. They also increase the expression of matrix metalloproteinases (MMPs), enzymes that break down collagen and aggrecan, leading to cartilage thinning, loss of joint function, pain, and inflammation [21] (Figure 1).

**Figure 2:**
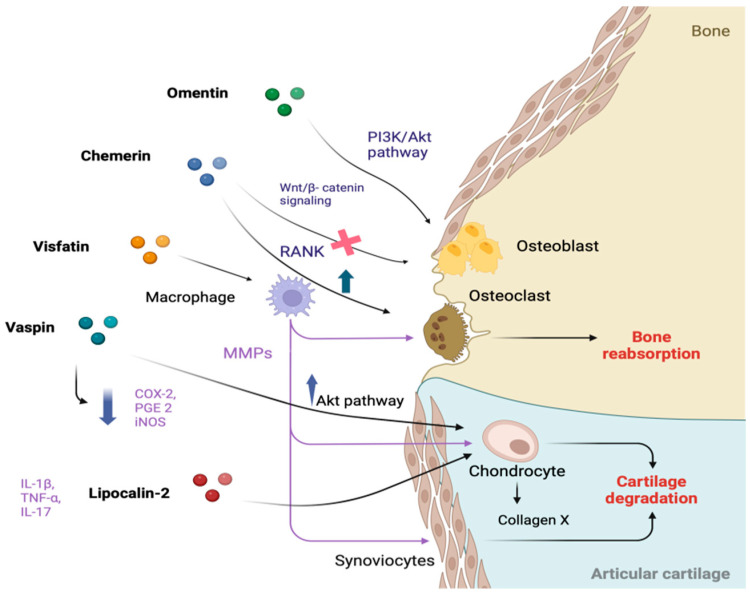
The role of minor adipokines in the pathogenesis of osteoarthritis with their pathways. Created with BioRender.com.

Additionally, adipokines influence subchondral bone metabolism, promoting sclerosis or increased remodeling, both characteristic of OA, driven by an imbalance between bone formation and resorption [22,23]. They also contribute to synovial inflammation by stimulating the release of pro-inflammatory mediators from synovial cells [24] (Figure 2).

Metabolic syndrome, often associated with obesity, is characterized by increased adipokine production. These metabolic disturbances can exacerbate OA [25] through systemic low-grade inflammation, contributing to joint damage even in non-weight-bearing joints, such as the hands and wrists. This suggests that obesity-related OA does not only have a mechanical origin [26,27,28].

Adipose tissue plays both a systemic and local role in OA pathogenesis. Recent studies have highlighted the role of the infrapatellar fat pad (IPFP), a fat deposit located between the knee joint capsule and the synovial membrane, which acts as an anatomical-functional unit with the synovial membrane [29]. The IPFP contributes to OA development through the production of pro-inflammatory cytokines and adipokines [30,31], exacerbating joint pain in affected patients [32]. Specifically, the IPFP in OA has been found to be more inflamed, vascularized, and fibrotic [33,34], with notably higher levels of leptin and chemerin in the IPFP and synovial tissues of OA patients compared to healthy controls [35].

**Figure 3:**
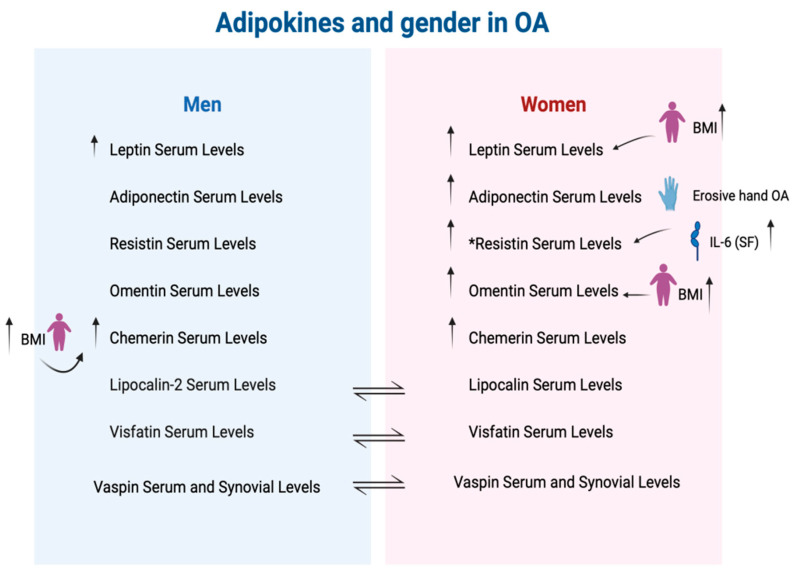
Adipokines, serum and synovial fluid levels, and gender differences. ↑: increased; ↑ (BMI): increasing related to BMI; ↑ (IL-6): increasing related to IL-6 sinovial fluid levels; *: Controversial result. Studies on the expression of resistin in osteoarthritis (OA) across genders have controversial results. A study by Presle did not find significant differences in the serum resistin levels between women and men [39]. In contrast, the research by Massengale et al. reported higher serum resistin levels in males (9.4 ng/mL) compared to females (7.3 ng/mL) [40].

Gender differences in adipokine levels reflect the complex interactions between sex hormones, fat distribution, and metabolic functions. Women typically exhibit higher serum levels of leptin [36], adiponectin [37], and omentin [38] than men (Figure 3). These differences contribute to distinct gender-related risks, not only for conditions like metabolic syndrome and cardiovascular disease but also for osteoarthritis.

The objective of our narrative review is to describe the mechanisms of action of key adipokines, their correlations between serum and synovial fluid levels, and their potential implications in OA pathogenesis. We focused on gender differences, synthesizing and critically evaluating the existing literature on how adipokines vary between men and women and how these differences influence the pathophysiology and progression of OA.

Overall, the review aims to provide a comprehensive understanding of how gender affects adipokine-related mechanisms in OA, offering insights that could lead to more effective and personalized treatments.

We conducted a narrative review, not adhering to PRISMA criteria. Our search was performed between February 2024 and April 2024 using the PubMed, MEDLINE, and Google Scholar databases, initially employing the MeSH terms “adipokines”, “gender”, and “osteoarthritis”. We included articles written in English and published until January 2024. Studies of all designs were considered, with preference given to systematic reviews and randomized controlled trials focused on the role of adipokines in osteoarthritis, particularly those highlighting gender differences (Table 1). Additionally, we included several references not identified by the search criteria but known to the authors or manually selected from the reference lists of screened articles. Abstracts without full-text access were excluded, as were articles not written in English.

## 2. Ke3. Adipokines Involved in the Development of Osteoarthritis

### 2.1. Leptin

#### 2.1.1. Structure, Function, and Physiology

Leptin is a 16 kDa non-glycosylated protein, primarily synthesized and secreted by mature adipocytes in white adipose tissue (WAT) [41]. Smaller amounts of leptin are also produced in other tissues, including brown adipose tissue (BAT), placenta, ovaries, skeletal muscle, brain, stomach, and pituitary gland. Leptin functions both as a hormone and a cytokine. As a hormone, it plays a crucial role in hematopoiesis, thermogenesis, angiogenesis, reproductive function [42], and weight regulation by suppressing food intake through a negative feedback mechanism [43]. As a cytokine, leptin promotes a low-grade pro-inflammatory state, increasing the risk of type II diabetes, cardiovascular events, and autoimmune disorders such as rheumatoid arthritis (RA) and systemic lupus erythematosus (SLE) [44].

Leptin is involved in both branches of the immune system. In the innate immune response, it stimulates the proliferation and phagocytosis of monocytes/macrophages; neutrophil chemotaxis; natural killer (NK) cell activation; and the secretion of pro-inflammatory cytokines (particularly TNF-α, IL-12, and IL-6) [45]. In adaptive immunity, leptin promotes a Th1 cell response, inhibiting Th2 cytokine production and the proliferation of regulatory T cells (T-reg) [46] while also increasing naïve T cells and IL-2 production via the mitogen-activated protein kinase (MAPK) and phosphatidylinositol 3-kinase (PI3K) pathways [47].

#### 2.1.2. Plasma and Synovial Fluid Concentration in OA

It has been demonstrated that plasma leptin concentrations are related to the percentage of body fat mass [48]. However, there is no consensus on the standard reference values. In a study by Considine et al., the mean (+/− SD) serum leptin concentrations were 31.3 +/− 24.1 ng/mL in obese subjects and 7.5 +/− 9.3 ng/mL in normal weight individuals [49].

Several studies have shown that leptin concentrations are higher in both the serum/plasma and synovial fluid of OA patients compared to healthy individuals [50]. It has also been established that synovial fluid and plasma leptin concentrations are positively related to the body mass index (BMI) [51].

Leptin levels in the synovial fluid can influence the joint microenvironment by promoting chondrocyte apoptosis through the upregulation of LOXL3 expression, inducing IL-6 and IL-8 expression and activating the OBRl/IRS-1/PI3K/Akt/AP-1 pathway [52]. Elevated leptin levels in synovial fluid also stimulate the secretion of metalloproteinases (MMP1 and MMP3), which contribute to the accelerated degradation of the extracellular matrix (ECM) [53].

#### 2.1.3. Clinical and Radiological Features of Leptin

A study by Bas et al. demonstrated that joint pain was associated with synovial fluid leptin levels in both hip and knee osteoarthritis (OA) [54]. The situation in hand osteoarthritis remains more uncertain: Massengale et al. found that serum concentrations of this adipokine were linked to pain intensity but not to radiographic severity; the most significant factors affecting joint damage were age and disease duration [40]. Similar findings were reported in the NHANES III cross-sectional study, which found no significant differences in the serum leptin levels among asymptomatic, symptomatic, and non-hand OA subjects [55].

In contrast, Morales Abaunza et al. observed a significant elevation in serum leptin concentrations in hand OA patients compared to healthy controls [56]. Additionally, the leptin levels were associated with the radiographic progression of cartilage damage, with higher plasma leptin levels found in patients at the more advanced third and fourth radiographic stages of knee osteoarthritis [57].

However, it remains unclear whether serum and SF leptin have distinct roles in the mechanisms involved in osteoarthritis.

#### 2.1.4. Differences between Genders

As previously mentioned, increased plasma concentrations of leptin in knee OA patients are observed in both sexes, with higher levels noted in females compared to males [58]. Additionally, BMI and female gender, both individually and in combination, show a strong positive correlation with leptin levels in synovial fluid among OA subjects. A comparative study by Hellström et al. found that, at each BMI level, including among lean individuals, women had significantly higher serum leptin values. The authors hypothesized that females have an increased production rate of leptin compared to males due to a less efficient leptin signaling system [59], which may explain the difference in body fat content between genders [60].

Indeed, while intra-abdominal fat accumulation correlates with insulin resistance, subcutaneous adipose tissue deposition is more strongly associated with circulating leptin levels [61]. A clinical trial by Bennett et al. confirmed that blood concentrations of leptin were independent of waist measurements in both men and women but were associated, especially in women, with hip circumference, a proxy for peripheral fat [62].

Sexual hormones play a significant role in regulating leptin production: testosterone has a suppressive effect [63], while estrogens act as stimulators. Rosenbaum et al. highlighted that premenopausal women had higher plasma leptin levels compared to postmenopausal women and men, even when adjusted for differences in body composition (premenopausal females > postmenopausal females > males) [64]. Moreover, many studies have reported significant fluctuations in leptin levels, with a steady increase from the lowest level during the early follicular phase to a peak during the late luteal phase [65], though this mechanism is still not fully understood.

### 2.2. Adiponectin

#### 2.2.1. Structure, Function, and Physiology

Adiponectin is a 28–30 kDa protein that is structurally homologous to collagen types VIII and X and complement component 1q (C1q) [66]. It is the most abundant adipokine synthesized in adipose tissue in various molecular forms but can also be produced by hepatocytes, epithelial cells, osteoblasts, myocytes, and pituitary cells [67]. Adiponectin exerts its biological effects through interaction with three cell surface receptors: AdipoR1, AdipoR2, and a smaller receptor known as T-cadherin.

AdipoR1 and AdipoR2 are part of the G protein-coupled receptor (GPCR) family and act as receptors for both the globular and full-length isoforms of adiponectin, mediating activities related to AMPK and PPAR-α ligands, as well as fatty acid oxidation and glucose uptake. AdipoR1 is predominantly expressed in the liver, skeletal muscles, macrophages, and hypothalamus, while AdipoR2 is found almost exclusively in the liver [68]. Ouchi et al. demonstrated that adiponectin suppresses TNF-alpha-induced IkappaB-alpha phosphorylation and subsequent NF-kB activation [69], reducing the transcription of genes involved in both innate and adaptive immune responses [70].

The third receptor, T-cadherin, is highly expressed in the cardiovascular system, especially in smooth muscle cells and endothelial cells across all types of blood vessels [71], and appears to play a protective role against neointimal and atherosclerotic plaque formation [72].

Numerous studies have confirmed the protective role of this adipokine against cardiovascular and metabolic diseases. The research by Ohashi et al. highlighted adiponectin’s function as a direct regulator of macrophage phenotypes, promoting a shift from a pro-inflammatory M1-like state to an anti-inflammatory M2-like state [73], suggesting its potential as a target for new therapeutic strategies.

#### 2.2.2. Plasma and Synovial Fluid Concentrations in OA

As previously noted for leptin, there is no international consensus on the reference range for adiponectin serum concentrations. Ahl et al. defined elevated levels of adiponectin as 10.46 μg/mL for men and 13.11 μg/mL for women among metabolically healthy individuals with a BMI of less than 25 kg/mq [74].

The relationship between serum and synovial fluid adiponectin levels and osteoarthritis (OA) remains a topic of debate. Chen et al. found that adiponectin levels in OA and synovial fluid were significantly lower compared to those in OA plasma [75]. Plasma levels of both total adiponectin and high molecular weight adiponectin (HMW) were also inversely related to the BMI, insulin resistance, and triglyceride levels, showing a decreasing trend in obesity that is reversible with weight loss [76,77].

Hu et al. were the first to report that adiponectin plays a protective role in chondrocytes by modulating autophagy through H2O2-induced apoptosis via the AMPK/mTOR signaling pathway [78]. Additionally, adiponectin binding to calreticulin/CD91 receptors enhanced the ability of macrophages to clear early opsonized apoptotic cells [79]. Synovial fluid adiponectin was significantly associated with the degradation markers of aggrecan, AGG1 and AGG2, indicating a potential role for adiponectin in regulating cartilage matrix degradation during OA progression [80].

Conversely, emerging research highlights a pro-inflammatory and catabolic role for adiponectin in OA pathogenesis. High adiponectin levels at the AdipoR1 level have been shown to stimulate inflammation through the AMPK and NF-κB signaling pathways, leading to the release of pro-inflammatory interleukins (such as IL-6), matrix metalloproteinases (MMP-1 and -3), and inducible nitric oxide synthase (iNOS), which contributes to pain, inflammation, and matrix degradation [81].

#### 2.2.3. Clinical and Radiological Features of Adiponectin

Plasma and synovial adiponectin levels have been positively associated with pain in patients with osteoarthritis [82]. Most studies on this relationship have focused on knee osteoarthritis, where adiponectin appears to be more closely linked to knee OA stiffness and soreness [83]. Furthermore, the leptin/adiponectin ratio in synovial fluid has been shown to be a more reliable local inflammatory marker than synovial adiponectin alone [84]. Higher concentrations of adiponectin in synovial fluid have also been associated with worse shoulder-specific pain scores in patients with shoulder OA [85].

The relationship between adiponectin levels and radiographic progression is controversial. Some studies have found that serum and synovial adiponectin levels are notably inversely related to the severity of radiographic osteoarthritis (ROA) [86,87] and negatively associated with the size of the osteophytes evaluated with MRI, suggesting a protective role against the onset of OA [88]. In contrast, other studies have highlighted that adiponectin levels are higher in patients with the most severe radiological OA [89], with a positive correlation between adiponectin concentration and Kellgren–Lawrence grading scores [90], suggesting a potential role as a mediator in cartilage degradation.

In the research by Xu et al., serum adiponectin levels were positively related to the radiographic severity of osteoarthritis in knee joints but not in hand joints, indicating different pathophysiological mechanisms in the development of OA among various joints [91].

#### 2.2.4. Differences between Genders

Christen et al. found that there are different plasma levels of adiponectin between sexes in OA patients, with women having significantly higher circulating levels than men [92]. A study by Orellana et al. also highlighted that, in women, synovial fluid adiponectin was more closely associated with clinical severity and the local inflammatory state in knee osteoarthritis than leptin [93]. Furthermore, research by Filkova et al. showed that, regardless of body mass index (BMI), female patients with erosive hand OA had higher serum adiponectin levels compared to those with a non-erosive pattern [94]. In contrast, similar analyses revealed a negative association between adiponectin and hand OA in men [95].

### 2.3. Resistin

#### 2.3.1. Structure, Function, and Physiology

Resistin, also known as adipose tissue-specific secretory factor (ADSF) or C/EBP-epsilon-regulated myeloid-specific secreted cysteine-rich protein (XCP1), is a 12.5 kDa cysteine-rich protein encoded by the RETN gene [96]. In humans, resistin exists in two isoforms: an oligomer with a molecular weight of 660 kDa and a smaller trimer of 45 kDa [97].

As resistin levels increase, its secondary structure tends to change. It is hypothesized that these molecular changes stabilize the resistin structure, making it resistant to denaturing environments [98] and biologically relevant for its activities [99].

This adipokine is found in various organs and tissues, with considerable variability among different species. In mice, it is almost exclusively expressed in the adipocytes of white adipose tissue and is related to preadipocyte differentiation [100]. In humans, however, only a small amount is synthesized by adipocytes, while the primary sources are leukocytes, including monocytes, macrophages, and neutrophils [101]. Recent studies have also detected small quantities in keratinocytes and sebocytes of the skin epidermis [102].

Resistin plays a significant role in glucose metabolism by inhibiting insulin-induced glucose uptake in myocytes through the downregulation of GLUT4 expression [103], leading to an increased risk of developing type 2 diabetes [104]. Resistin levels are influenced by the circadian rhythm, which presents an opposite pattern compared to insulin [105]. Insulin resistance is closely associated with obesity and visceral fat accumulation. Resistin is considered a key factor linking these conditions [106].

Additionally, resistin regulates the production of pro-inflammatory cytokines such as TNF-alpha, IL-6, and IL-12 in macrophages, which are involved in OA pathogenesis, via the NF-kB-dependent pathway [107]. Toll-like receptor 4 (TLR4) has also been proposed as a mediator of resistin-induced secretion of pro-inflammatory factors [108]. These interactions help explain resistin’s multifunctional role in chronic inflammation, atherosclerosis, and insulin resistance.

#### 2.3.2. Plasma and Synovial Fluid Concentrations in OA

In healthy individuals, normal serum levels of resistin range from 7 to 22 ng/mL [109]. Studies have shown that this adipokine can influence bone and cartilage metabolism by upregulating the expression of cytokines and matrix-degrading enzymes through the p38-MAPK and NF-κB signaling pathways in human chondrocytes [110]. This upregulation leads to increased pro-apoptotic effects [111] and superoxide anion production [112]. Resistin serum levels have also been positively associated with changes in the joint microenvironment, including increased signal intensity in the infrapatellar fat pad (IPFP), effusion synovitis, and bone marrow lesions [113]. Additionally, resistin significantly affects the type I collagen composition of subchondral bone, shifting the phenotype of normal weight bone towards a sclerotic, homotrimer-rich type I collagen phenotype characteristic of overweight or obese bone [114]. No significant associations have been found with cartilage volume loss.

#### 2.3.3. Clinical and Radiological Features of Resistin

A study by Song highlighted that, in knee osteoarthritis (OA) patients, only resistin concentrations in synovial fluid were significantly associated with pain, physical function, Kellgren–Lawrence grades, and levels of C-telopeptides of type II collagen, a marker of osteoclast activity [115]. These findings were confronted with those that emerged from the study by Calvet et al., which found that synovial fluid resistin was more closely related to joint dysfunction and stiffness than to pain [116].

In line with these results, some authors have noted that, in hand OA patients, circulating levels of resistin are positively associated with leptin levels [84] and correlate with radiographic changes, especially with subchondral erosion [117]. However, other studies did not find an association between leptin and resistin levels and the progression of hand OA [91,118].

Moreover, high levels of resistin are not exclusive to osteoarthritis. Lee et al. observed increased resistin concentrations in synovial fluid and serum samples from patients with knee injuries (such as anterior cruciate ligament or meniscus tears)—in particular, within the first week after the traumatic event [119].

Additionally, resistin was found at higher levels in the synovium tissue of rheumatoid arthritis patients compared to those with spondyloarthritis or osteoarthritis, and it was directly related to both CRP values and the DAS28 measure [120]. Thus, larger and more standardized studies are needed to confirm the potential role and biological effects of this adipokine.

#### 2.3.4. Differences between Genders

Studies on the expression of resistin in osteoarthritis (OA) across genders have yielded controversial results. A study by Presle did not find significant differences in the serum resistin levels between women and men [39]. In contrast, the research by Massengale et al. reported higher serum resistin levels in males (9.4 ng/mL) compared to females (7.3 ng/mL) [40].

Additionally, a significant positive correlation was observed between synovial fluid levels of IL-6 and resistin in women but not in men [121,122]. Furthermore, a negative correlation was found between resistin levels and pain intensity in men with OA [123].

## 3. Other Minor Adipokines Involved in Osteoarthritis

### 3.1. Chemerin

#### 3.1.1. Structure, Function, and Physiology

Chemerin, also known as retinoic acid receptor responder protein 2 (RARRES2) or tazarotene-induced gene 2 protein (TIG2), is a 16 kDa protein primarily expressed in white adipose tissue [124]. It is initially synthesized in an inactive form (prochemerin) consisting of 63 amino acids, which undergoes proteolytic cleavage at its C-terminus by proteases derived from neutrophils (elastase and cathepsin G), mast cells (tryptase), the coagulation cascade, and some bacterial enzymes [125]. This adipokine exerts its effects by binding to several G protein-coupled receptors, including ChemR23 (also known as CMKLR1), GPR1 (G-protein coupled receptor 1), and CCRL2 (C-C chemokine receptor-like 2) [126]. These receptors are found in activated monocytes/macrophages; natural killer (NK) cells; foam cells; and various tissues such as liver, intestine, kidneys, endothelial cells, and adipose tissue [127], suggesting a mediating role between the immune and metabolic systems.

In the immune system, chemerin acts as a chemoattractant adipokine, promoting the migration of dendritic cells and macrophages to inflammation sites while also stimulating neoangiogenesis by enhancing endothelial cell proliferation through the expression of vascular endothelial growth factor (VEGF) and adhesion molecules (ICAM and E-selectin) [128]. Regarding energy metabolism, chemerin plays a crucial role in promoting lipolysis and the differentiation of pre-adipocytes into mature cells [129]. Its role in regulating glucose uptake and insulin sensitivity is somewhat controversial, as it has been shown to both promote insulin signaling [130] and downregulate insulin-stimulated glucose uptake [131].

#### 3.1.2. Role of Chemerin in OA and Differences between Genders

Chemerin can be detected in serum, cartilage, and synovial fluid, with ChemR23 found in human chondrocytes, where it enhances the activity of matrix metalloproteinases (MMPs) [132]. However, the precise mechanisms by which chemerin influences bone metabolism and osteoarthritis (OA) remain a matter of debate. It has been shown that chemerin inhibits osteoblast differentiation and proliferation by suppressing Wnt/β-catenin signaling while stimulating osteoclast differentiation and proliferation via RANK signaling, suggesting its involvement in the pathogenesis of osteoporosis [133].

Clinical studies examining the relationship between chemerin levels and OA are limited, with the results showing considerable variability. Elevated chemerin levels in both synovial fluid (SF) and serum have been observed in patients with knee OA, correlating with increased concentrations of inflammatory markers such as C-reactive protein (CRP), TNF-α, and interleukin-6 (IL-6), indicating a potential link between chemerin and the inflammatory mechanisms characteristic of OA [134].

Huang et al. reported significantly higher synovial chemerin levels in knee OA (KOA) patients with Kellgren–Lawrence (KL) grade 4 compared to those with KL grades 2 and 3, suggesting a positive correlation between chemerin levels and knee OA severity [135]. Similarly, Ma et al. found elevated levels of this adipokine in the synovial fluid of knee OA patients compared to a control group [136].

In a study of Colombian patients with primary osteoarthritis, Cajas Santana et al. observed that the chemerin levels averaged 373,525 ng/mL in OA patients compared to 17,555 ng/mL in healthy controls. However, they found no correlation between chemerin levels and the number of affected joint regions (especially the hands and knees), disease severity according to the K/L radiological scale, or body mass index (BMI) [137].

In support of the variability in these findings, Philip et al. reported significantly higher circulatory chemerin levels in patients with end-stage hip OA compared to lean controls, independent of the BMI. Despite this, no significant increase in serum chemerin levels was observed in hip OA patients in relation to disease severity, including KL grade, joint space narrowing, or osteophytes formation [138].

The role of sex hormones in chemerin production and regulation remains largely unexplored. In humans, serum chemerin expression has been found to be significantly higher in women than men. Conversely, in the study of Takahashi et al., higher chemerin levels were measured in males with metabolic syndrome compared to females [130]. However, Stejskal et al. found no significant sex differences in serum chemerin levels in healthy controls, highlighting the need for further investigations [139].

Thus, no significant correlations have been identified between chemerin levels in SF and age or gender in OA patients.

### 3.2. Visfatin

#### 3.2.1. Structure, Function, and Physiology

Visfatin, also known as pre-B cell colony-enhancing factor 1 (PBEF1) or nicotinamide phosphoribosyl transferase (NAmPRTase or Nampt), is a 52 kDa adipokine primarily synthesized by adipocytes, particularly in visceral rather than subcutaneous tissue [140]. However, it is also expressed in skeletal muscles, liver, bone marrow, lymphocytes, and macrophages [141].

While the receptor for visfatin remains unidentified, four distinct signaling pathways have been linked to its activity. The first pathway is mediated by β1 integrin and triggers signaling through the ERK, p38 MAPK, NF-kB, and AP-1 pathways. The second involves IL-6, STAT3, NAMPT, Sirt-1, and Sirt-6. The third is characterized by redox mechanisms, enhancing the activity of antioxidant enzymes like superoxide dismutase (SOD), catalase (CAT), and glutathione peroxidase (GSHPx). The fourth pathway is mediated by the insulin receptor (IR) and involves the PI3K, Akt, MAPK, and ERK1 pathways [142].

Several studies have shown a potential positive correlation between circulating visfatin levels and various anthropometric or metabolic parameters in individuals with visceral obesity, type 2 diabetes, metabolic syndrome, and cardiovascular diseases [143,144,145].

In vitro research has demonstrated that visfatin can stimulate the expression of pro-inflammatory cytokines (IL-1β, IL-6, IL-10, TNF-α, and IL-17) in human monocytes [146]. Additionally, when combined with IL-7, visfatin promotes B cell maturation, stimulates VEGF expression, activates MMP2 and MMP9, and inhibits neutrophil apoptosis [147]. In osteoarthritis (OA) synovial cells stimulated by visfatin and resistin, an increase in MMP-1 and MMP-13 expression, along with a decrease in Col2a1 expression, has been observed [111].

These findings suggest that visfatin may act as a pro-inflammatory mediator, particularly under conditions related to anthropometric or metabolic factors, especially in males [148].

#### 3.2.2. Role of Visfatin in OA and Differences between Genders

Patients with osteoarthritis (OA) exhibit higher levels of visfatin compared to healthy individuals, with visfatin being predominantly expressed in synovial tissue, chondrocytes, osteophytes, osteoblasts, and osteoclasts [149]. Fioravanti et al. observed elevated serum visfatin levels in hand osteoarthritis (HOA), particularly in erosive HOA compared to non-erosive cases [150]. In OA patients, visfatin promotes cartilage matrix degradation through multiple mechanisms: it inhibits the expression of key factors essential for maintaining the chondrocyte phenotype, such as SOX9 and type II collagen [151], facilitates the release of metalloproteases [152], and reduces the synthesis of high-molecular-weight proteoglycans [153]. Cheleschi et al. confirmed that visfatin induces apoptosis and oxidative stress in cultured human OA chondrocytes by increasing the expression of miR-34a and miR-181a via the NF-κB pathway [154]. These microRNAs are involved in signaling pathways regulating chondrocyte proliferation and apoptosis during OA progression.

These findings align with studies showing a positive correlation between increased synovial fluid (SF) visfatin levels in OA patients and higher concentrations of cartilage degradation biomarkers, such as C-terminal telopeptide of type II collagen (CTX-II) [155]. It has also been observed that the joint microenvironment influences synovial visfatin levels. The infrapatellar fat pad (IFP) releases larger amounts of this adipokine compared to subcutaneous adipose tissue [156], and osteophytes are another significant source of visfatin in OA-affected joints [157].

Further supporting evidence comes from Chen et al., who found elevated visfatin concentrations in knee OA patients compared to controls, with higher levels present in the synovial fluid than in paired plasma samples [158]. Comparing visfatin with adiponectin and resistin in knee OA patients, Calvet et al. demonstrated that visfatin, similar to resistin, is more strongly associated with functional impairment, whereas adiponectin is primarily linked to pain [116].

Reviewing the literature, no significant difference between sexes was observed. Although Chen et al. reported higher visfatin levels in the serum and synovial fluid of women with OA compared to men, this difference was not statistically significant [158]. Additionally, an increase in visfatin levels was noted during flare-ups in knee OA patients, with the levels subsequently decreasing during remission, with no variations when adjusted for age or gender [159].

### 3.3. Omentin

#### 3.3.1. Structure, Function, and Physiology

Omentin, also known as omentin-1 (the predominant form) or intelectin-1, is a protein composed of 313 amino acids primarily found in human visceral and subcutaneous fat, with lower expression in the small intestine. Recent research has highlighted its anti-inflammatory effects in vascular endothelial cells, where it prevents TNF-α-induced COX-2 expression by inhibiting the JNK signaling pathway, presumably through the activation of the AMPK/eNOS/NO pathways [160]. In healthy individuals, circulating omentin levels show a negative correlation with obesity-related parameters such as BMI, waist circumference, and leptin concentration [161].

Omentin has also been studied as a marker of disease activity in certain autoinflammatory disorders. Lu et al. demonstrated that serum omentin-1 levels were negatively correlated with disease activity in Crohn’s disease, offering a better marker than C-reactive protein (CRP) [162]. Additionally, higher serum omentin levels have been observed in patients with juvenile idiopathic arthritis compared to healthy controls, with a significant correlation to inflammatory disease activity [163].

However, it remains unclear why omentin-1 levels are significantly downregulated in various conditions like obstructive sleep apnea, pancreatitis, psoriasis, obesity, insulin resistance, diabetes mellitus, and ischemic heart disease while being upregulated in others, such as liver cirrhosis, non-alcoholic steatohepatitis, and lupus nephritis [164,165].

#### 3.3.2. Role in OA and Differences between Genders

Recent evidence has shown that omentin reduces the release of inflammatory cytokines such as IL-6 and TNF-α while promoting the proliferation of human osteoblasts through the PI3K/Akt pathway [166]. Omentin has been proposed as an independent predictor of bone mineral density, potentially preventing inflammation-induced osteoporosis by downregulating pro-inflammatory cytokines; however, further studies are required to confirm this hypothesis [167].

Serum levels of omentin-1 have been observed to be similar between patients with knee osteoarthritis (OA) and healthy individuals, although its concentrations in synovial fluid (SF) were significantly lower in OA patients and decreased with disease severity, as measured by Kellgren–Lawrence (KL) grades, particularly in obese patients [168]. Ko et al. demonstrated that omentin induces IL-13- and IL-4-dependent anti-inflammatory responses and promotes M2 macrophage polarization in OA synovial fibroblasts via the PI3K, ERK, and AMPK pathways, thereby reducing osteoarthritis progression [169]. This adipokine also inhibits the expression of MMP-1, MMP-3, and MMP-13, which are mediated by the inflammatory cytokine interleukin-1β (IL-1β) in human chondrocytes, both at the mRNA and protein levels, contrasting progressive cartilage degradation [170].

De Souza Batista et al. reported that serum omentin concentrations, as well as omentin expression in visceral adipose tissue, were significantly lower in overweight and obese individuals compared to lean ones [171]. After adjusting for body mass index (BMI), women were found to have higher circulating levels of omentin-1 than men. This finding aligns with a study by Tan et al., which showed that omentin mRNA expression correlates negatively with 17β-estradiol [172].

In summary, serum omentin-1 levels are inversely correlated with BMI, waist circumference, leptin levels, and insulin resistance while positively correlated with adiponectin and HDL levels. Omentin-1 thus presents promising therapeutic potential for protecting cartilage health, modulating inflammation, and improving mitochondrial performance in chondrocytes in OA. It also appears to be related to the bone turnover, as Dikker et al. found higher omentin-1 levels in postmenopausal women with osteoporosis compared to premenopausal women, suggesting a positive correlation with vitamin D, possibly due to its direct effect on adipose tissue [173].

Lastly, sex differences in OA related to omentin-1 levels remain to be explored.

### 3.4. Lipocalin-2

#### 3.4.1. Structure, Function, and Physiology

Lipocalin-2 (LCN2), also known as siderocalin, uterocalin, or neutrophil gelatinase-associated lipocalin (NGAL), is a 25 kDa glycoprotein primarily produced in white adipose tissue. It exists in three different isoforms: a 25 kDa monomer, a 46 kDa homodimer, and a 135 kDa heterodimer complexed with MMP9 in human neutrophils [174].

This adipokine interacts with at least two surface receptors: the LCN2 receptor (also known as 24p3R, NGALR, or SLC22A17), a brain-type organic cation transporter (BOCT), and megalin (also known as low-density lipoprotein receptor-related protein 2, LRP2, or gp330), a multiligand scavenger receptor [175].

Lipocalin-2 is believed to bind small molecules such as steroids and lipopolysaccharides, and it has been reported to play roles in the induction of apoptosis in hematopoietic cells, the transport of fatty acids and iron, and inflammatory modulation [176]. It has also been shown to contribute to metabolic homeostasis, being positively associated with adiposity, hyperglycemia, insulin resistance, and CRP levels [177].

#### 3.4.2. Role in OA and Differences between Genders

Lipocalin-2 (LCN2) is expressed in joint tissues and functions as a mechanosensitive adipokine [178]. Its expression in osteoblasts is upregulated by pro-inflammatory cytokines such as IL-1β, TNF-α, and IL-17 in the absence of mechanical force stimulation, while TGF-β1 and IGF-1 act as inhibitors [179]. In joints, LCN2 promotes the synthesis of type X collagen and supports chondrocyte differentiation and proliferation [180].

Patients with osteoarthritis (OA) have been found to have elevated levels of neutrophil gelatinase-associated lipocalin (NGAL) compared to healthy individuals, although these levels are lower than those observed in rheumatoid arthritis (RA) patients. However, Maurizi et al. mitigates the relevance of LCN2 as a serum biomarker for OA, reporting no significant differences between healthy and osteoporotic women [181]. Moreover, a study with mouse models showed that LCN2 alone is neither necessary nor sufficient to induce cartilage damage in post-traumatic OA [182]. Instead, Lim WH et al. found that circulating LCN2 levels are predictive of future osteoporotic fractures requiring hospitalization [183].

Similar to omentin, lipocalin-2 plasma concentrations, associated with cardiometabolic risk factors such as an increased triglycerides-to-high-density lipoprotein (HDL) cholesterol ratio [184], do not exhibit a gender-specific pattern in OA patients, although, in healthy individuals, its circulating levels appear to be age- and gender-related.

### 3.5. Vaspin

#### 3.5.1. Structure, Function, and Physiology

Vaspin is a 50 kDa adipokine, also known as a visceral adipose tissue-derived serine protease inhibitor, primarily produced by visceral fat. It is mainly known for its insulin-sensitizing properties and its role in regulating glucose tolerance. Reduced levels of vaspin have been observed in several conditions compared to healthy individuals, including type II diabetes, metabolic syndrome, obesity, and coronary artery disease [185].

There have only been a few studies on its molecular mechanism of action. Phalitakul et al. demonstrated that, during inflammatory episodes involving vascular smooth muscle cells, vaspin inhibits TNF-α-induced expression of intracellular adhesion molecule (ICAM-1) by preventing the generation of reactive oxygen species (ROS) and subsequent activation of NF-κB and protein kinase C (PKC) [186].

#### 3.5.2. Role in OA and Differences between Genders

A study by Bao et al. revealed that joint tissues such as cartilage, synovium, meniscus, infrapatellar fat pad, and osteophytes express the vaspin gene in patients with osteoarthritis (OA). Additionally, vaspin protein was detected in cartilage, synovium, and osteophytes. Serum vaspin levels were higher in OA patients compared to healthy individuals, and vaspin concentrations were higher in serum than in synovial fluid (SF) [187]. Another study by Bao et al. highlighted that low vaspin levels reduced the IL-1β-induced expression of catabolic molecules, such as MMP-2, MMP-9, ADAMTS-5, and cathepsin D. This study also demonstrated that vaspin could inhibit IL-1β-stimulated production of inflammatory mediators, including COX-2, PGE2, and iNOS, in a dose-dependent manner in chondrocytes, suggesting that vaspin has anti-catabolic and anti-inflammatory properties [188]. Additionally, vaspin may enhance the chondrogenic differentiation of bone marrow mesenchymal stromal cells and stimulate the proliferation and extracellular matrix (ECM) production in chondrocytes through activation of the Akt pathway, thereby protecting chondrocytes during flogistic conditions [189].

He et al. underscored vaspin’s role in inhibiting miR-155 expression in rat chondrocytes and promoting cholesterol efflux. When vaspin expression is reduced, LXRα and other genes involved in cholesterol efflux are suppressed in chondrocytes, leading to cholesterol accumulation, which can worsen the progression of OA [190].

Serum or synovial fluid vaspin levels do not appear to correlate with age or sex in obese patients with type II diabetes [191], and its role in gender-related differences in OA remains to be explored.

## 4. Discussion

In this review, we aim to provide a comprehensive and practical update for physicians interested in this field (Table 2). The main findings from the literature highlight the association between osteoarthritis (OA) and elevated serum and synovial levels of leptin, chemerin, visfatin, and high plasma levels of resistin.

These observations suggest different roles for these molecules and their potential contribution to the pathogenesis of OA.

Specifically, leptin promotes cartilage degradation by inducing IL-6 and IL-8 expression, activating the OBR1/IRS-1/PI3K/Akt/AP-1 pathway [52], and stimulating the secretion of metalloproteinases (MMP1 and MMP3) [53]. Chemerin contributes to determine a catabolic bone environment by inhibiting osteoblast differentiation and proliferation through suppression of Wnt/β-catenin signaling while promoting osteoclast differentiation via RANK signaling [192]. In OA patients, visfatin contributes to cartilage matrix breakdown by suppressing the factors essential for maintaining a chondrocyte phenotype, such as SOX9 and type II collagen [151], triggering metalloproteases release [152], and decreasing the production of high-molecular-weight proteoglycans [153]. Resistin is involved in bone and cartilage metabolism by increasing the expression of cytokines and matrix-degrading enzymes through p38-MAPK and NF-κB signaling pathways [110], and its serum levels are associated with alterations in the joint microenvironment, including changes in the infrapatellar fat pad, effusion synovitis, and bone marrow lesions [113].

Conversely, the actions of different adiponectin forms can be highly variable. Adiponectin and omentin levels in synovial fluid are reduced in OA patients compared to healthy subjects, with an inverse relationship to disease severity, which may indicate their protective roles. Omentin, in particular, decreases the release of inflammatory cytokines such as IL-6; stimulates osteoblast proliferation through the PI3K/Akt pathway [166]; promotes M2 macrophage polarization in osteoarthritis synovial fibroblasts [169]; and inhibits the expression of MMP-1, MMP-3, and MMP-13, helping to prevent further cartilage degradation [170], potentially contrasting OA progression.

Adiponectin presents both protective and damaging effects in OA. It protects chondrocytes by regulating autophagy through the AMPK/mTOR signaling pathway during H2O2-induced apoptosis [78] and enhances macrophages’ ability to remove early apoptotic cells through calreticulin/CD91 receptors [79]. Its levels in synovial fluid were significantly associated with the degradation markers of aggrecan, AGG1 and AGG2, suggesting that adiponectin may also be involved in the regulation of cartilage matrix degradation during the progression of osteoarthritis [80]. However, recent studies have also highlighted its pro-inflammatory and catabolic effects in OA, especially in relation to gender. Elevated adiponectin levels, acting through the AdipoR1 receptor, promote inflammation via the AMPK and NF-κB signaling pathways, leading to the release of pro-inflammatory interleukins like IL-6, matrix metalloproteases (MMP-1 and MMP-3), and inducible nitric oxide synthase (iNOS), contributing to pain, inflammation, and matrix degradation [81]. Women tend to have higher circulating adiponectin levels than men [75,92], with greater clinical severity independent of body mass index (BMI) and a more pronounced local inflammatory state [93,94].

It is noteworthy that there is a positive correlation between the leptin and resistin levels with joint pain and radiographic progression, while adiponectin’s effects appear to be more related to the clinical aspects of the disease.

Given the growing interest in exploring gender differences and the relationship between gender/sex and OA, as well as the limited information on differential biomarker expressions, we have extracted key insights from the analyzed studies. The data show that female OA patients have higher serum levels of leptin, chemerin, and omentin compared to males, with a positive correlation with the BMI and estrogen levels. This provides a molecular explanation for the sexual dimorphism observed in OA.

Regarding visfatin and lipocalin, studies have not revealed significant differences in the synovial and serum levels between sexes. However, findings on resistin remain controversial, with some studies showing no sex differences and others demonstrating higher values in women and a positive relationship with the IL-6 levels.

Some studies suggest that rehabilitation treatments might influence the serum and synovial levels of adipokines. We hope that future research on adipokines will lead to treatments that can locally target these molecules in OA, offering optimal results with fewer systemic effects.

## 5. Conclusions

Gender medicine seeks to understand how biological sex and gender influence health, disease progression, and treatment outcomes. Adipokines, bioactive proteins secreted by adipose tissue, play a crucial role in metabolic regulation and inflammatory processes, including conditions like osteoarthritis (OA) and other metabolic diseases.

Many molecular pathways contribute to predisposition, onset, and phenotypic characterization among OA patients. The partial exhaustiveness of the current data underscores the need for further research to fully elucidate the role of each adipokine in the development and progression of osteoarthritis.

Among the aspects still not well understood are the mechanisms of interaction between the metabolic, mechanical, and immune systems in cases of altered bone and cartilage metabolism. We believe this area of molecular research offers a valuable opportunity for future advancements in diagnosis and the identification of new biological markers, as well as potential targets for managing this degenerative disease.

Novel therapies tailored to each patient’s profile, specifically targeting adipokines, present a promising avenue for OA treatment. For example, leptin inhibitors could help prevent leptin-mediated cartilage degradation, especially in postmenopausal women with elevated leptin levels. Similarly, adiponectin-mimetic drugs could be developed to enhance its protective effects in both genders, particularly for individuals with metabolic syndrome.

Further investigation into the hormonal and metabolic regulation mechanisms of adipokines in connection with joint health is needed to uncover new possibilities for therapeutic interventions.

## Figures and Tables

**Table 1:** Data collected.

Adipokine	Number of Studies	Interval Period	Sites of OA
Leptin	43	1996–2024	Knee (15) Hand (6) Hip (2)
Adiponectin	45	2000–2024	Knee (21) Hand (8) Hip (3) Shoulder (2)
Resistin	39	2001–2024	Knee (13) Hand (5) Hip (4) Shoulder (1)
Chemerin	18	2005–2024	Knee (5) Hand (1) Hip (1)
Visfatin	25	2006–2024	Knee (8) Hand (1) Hip (1)
Omentin	10	2007–2024	Knee (3)
Lipocalin–2	9	2005–2024	Knee (1)
Vaspin	5	2011–2024	Knee (1)

**Table 2:** The relationships between adipokine levels and various aspects of metabolism, osteoarthritis, and gender have been examined. These relationships are illustrated as follows: in sky blue for synovial fluid, in red for plasma, and in green for both plasma and synovial fluid.

Adipokine	Role	Metabolism	OA Joint Pain	OA Radiological Severity	Gender
Leptin	Pro– Inflammatory	High BMI Sub -cutaneous fat	Hip Knee	Knee	Female
Adiponectin	Anti– inflammatory (metabolism) Pro– inflammatory (OA)	Weight loss	Knee	///	Female
			Shoulder		
Resistin	Pro–inflammatory	Obesity	Knee	Knee	///
Chemerin	Pro–inflammatory	High BMI	///	Knee	///
Visfatin	Pro– inflammatory	High BMI Visceral fat	Knee	Knee	///
			Hand	Hand	
Omentin	Anti– inflammatory	High BMI Visceral fat (negative relationship)	Knee (negative relationship)	Knee (negative relationship)	Female
Lipocalin-2	Pro– inflammatory	Dyslpidemia	///	///	///
Vaspin	Pro–inflammatory	Metabolic syndrome	Knee	Knee	///

## Abbreviations

The table below provides the definitions of various abbreviations and acronyms mentioned in this paper.

**Abbreviation**	**Meaning**
ABCA1	ATP-binding cassette transporter
ADAMTS	Disintegrin and metalloprotease with thrombospondin motifs
ADSF	Adipose Tissue-Specific Secretory Factor
AMPK	AMP-Activated Protein Kinase
BMI	Body Mass Index
CCRL2	Chemokine C-C motif receptor
ChemR23	Chemerin and resolvin E1 receptor
CMKLR1	Chemokine-like receptor 1
CTX-II	C-terminal telopeptide of type II collagen
ECM	Extracellular matrix
ERK	Extracellular signal-regulated kinase
GLUT	Glucose transporter
GPCRs	G protein-coupled receptors
HOA	Hand osteoarthritis
ICAM	Intracellular adesion molecule
IGF	Insultin-like growth factor
IPFP	Infrapatellar fat pad
iNOS	Inducible nitric oxide synthase
KOA	Knee osteoarthritis
LCN2	Lipocalin-2
LES	Lupus erythematosus systemicus
LOXL	Lysyl oxidase omolog
MAPK	Mitogen-activated protein kinases
MMP	Matrix metalloproteinase
MRI	Magnetic resonance imaging
mTOR	Mechanistic target of rapamicin
NAMPT	Nicotinamide phosphoribosyl transferase
NF-kB	Nuclear factor kappa light chain enhancer of activated B cells
OA	Osteoarthritis
PI3K	Phosphatidylinositol 3 kinase
PPAR	Peroxisome proliferator activated teceptors
RA	Rheumatoid arthritis
RANK	Receptor activator of nuclear factor-kb
RARRES2	Retinoic acid receptor responder protein 2
ROA	Radiographic osteoarthritis
SpA	Spondyloarthritis
SF	Synovial fluid
STAT	Signaling transducer and activator of transcription
TGF	Transforming growth factor
TIG2	Tazarotene-induced gene 2 protein
TLR	Toll-like receptor
TNF	Tumor necrosis factor
VEGF	Vascular endotelial growth factor
XCP1	C/EBP-epsilon-regulated myeloid-specific secreted cysteine-rich protein
